# Nature, nurture, and expertise

**DOI:** 10.1016/j.intell.2013.06.008

**Published:** 2014-07

**Authors:** Robert Plomin, Nicholas G. Shakeshaft, Andrew McMillan, Maciej Trzaskowski

**Affiliations:** King's College London, MRC Social, Genetic & Developmental Psychiatry Centre, Institute of Psychiatry, London, SE5 8AF, United Kingdom

**Keywords:** Twins, Reading, Genotype–environment interaction and correlation, Non-shared environment, Liability-threshold

## Abstract

Rather than investigating the extent to which training can improve performance under experimental conditions (‘what could be’), we ask about the origins of expertise as it exists in the world (‘what is’). We used the twin method to investigate the genetic and environmental origins of exceptional performance in reading, a skill that is a major focus of educational training in the early school years. Selecting reading experts as the top 5% from a sample of 10,000 12-year-old twins assessed on a battery of reading tests, three findings stand out. First, we found that genetic factors account for more than half of the difference in performance between expert and normal readers. Second, our results suggest that reading expertise is the quantitative extreme of the same genetic and environmental factors that affect reading performance for normal readers. Third, growing up in the same family and attending the same schools account for less than a fifth of the difference between expert and normal readers. We discuss implications and interpretations (‘what is inherited is DNA sequence variation’; ‘the abnormal is normal’). Finally, although there is no necessary relationship between ‘what is’ and ‘what could be’, the most far-reaching issues about the acquisition of expertise lie at the interface between them (‘the nature of nurture: from a passive model of imposed environments to an active model of shaped experience’).

## Highlights

• More than half of the difference between expert and normal readers is genetic.
• Expert readers show the same genetic effects as normal readers.
• Less than a fifth of the expert-normal difference is due to shared environment.
• Passive models of training regimes imposed on children address ‘what could be’.
• Active models of selected environments will foster the acquisition of expertise.

## 1 Introduction

The relative influence of nature and nurture has been central to research on expertise since Francis Galton's (1865) two-article series on hereditary genius, which he expanded into the first book in the field of behavioral genetics, *Hereditary genius: an inquiry into its laws and consequences* (Galton, 1869). Using mere reputation as an index, Galton suggested that ability – brains as well as brawn – runs in families. He greatly overinterpreted his results to conclude that genius is hereditary and that “ability will out” regardless of environment.

During the 150 years since Galton's first papers, the pendulum has swung back and forth between nature and nurture in the behavioral sciences. For the first fifty years, the influence of Galton and his cousin, Charles Darwin, pushed the pendulum towards nature. In the 1920s, John Watson's behaviorism, which led to environmentalism, began to push the pendulum towards nurture. This swing was accelerated by the eugenic horrors of Nazi Germany in the 1930s and 1940s. After World War II, psychology was dominated by learning theory and an environmentalism that assumed that we are what we learn. However, by the 1960s and 1970s, the pendulum began to swing back towards a more balanced view that recognized the importance of nature as well as nurture. With the breath-taking advances in genetics in recent years, there is some danger now that the pendulum may be swinging too far back to nature (Plomin, 2013).

In all areas of the behavioral sciences, genetic influence has been shown to account for substantial variance, but this same research provides strong evidence for the importance of environment as well. Heritability, which is an effect size index of the proportion of phenotypic variance that is accounted for by genetic variance, is typically between 30 and 60% across psychological traits, which means that 40–70% of the variance is *not* genetic in origin (Plomin, DeFries, Knopik, & Neiderhiser, 2013). The issue is not nature versus nurture, but rather nature *and* nurture because both are important, which suggests that the way forward is to develop strategies that bring nature and nurture together to help us understand the development of complex traits (Rutter, Moffitt, & Caspi, 2006). There are signs that the nature–nurture battles are over. For example, over 90% of parents and teachers of young children believe that nature is as important as nurture in the development of a wide range of behavioral traits, including intelligence, learning disabilities, personality and mental illness (Walker & Plomin, 2005).

In this context, the domain of expertise might seem atavistic, stuck in the nature versus nurture era. However, this view is more apparent than real because the extreme environmentalist position has been promoted by very few people (Ericsson, 2007; Howe, Davidson, & Sloboda, 1998); in the other corner, we can find no one who espouses an extreme hereditarian position. If a survey of opinions about the relative importance of nature and nurture in expertise were conducted in academia like the one mentioned above for parents and teachers, we predict that academics in all disciplines would also overwhelmingly accept the importance of nature as well as nurture. (See Tucker & Collins, 2012, in relation to sporting success.) In our opinion, this faux debate about nature *versus* nurture in the domain of expertise is a distraction that obscures many interesting empirical questions about the origins of expertise.

In this paper, we consider expertise as exceptional performance (Simonton, 2011), ignoring semantic and etymological issues about words such as ‘talent’ and ‘genius’. People who excel can of course be found in any domain of performance, such as music, athletics, games, and cognitive performance. The topic of this special issue is the acquisition of expertise, which we interpret as asking why some people become experts and others do not. It is important to understand the origins of expertise as it exists in the real world of sports, arts and skills. We refer to the origins of such real-world expertise as ‘what is’ in order to contrast this approach to much research on the acquisition of expertise that asks a different question about ‘what could be’ — investigating the extent to which expertise can be acquired by intensive training and practice. The critical point is this: There is no necessary connection between ‘what is’ and ‘what could be’. That is, even if the difference between experts' performance and the performance of the rest of the population were due solely to genetic differences (what is), a new environmental intervention such as a new training regime could still greatly improve performance (what could be). For example, although obesity is highly heritable, if people stop eating they will lose weight; moreover, a novel environmental intervention such as bariatric surgery can dramatically reduce extreme obesity (Dixon, Straznicky, Lambert, Schlaich, & Lambert, 2011). Showing that diets and other interventions can make a difference (what could be) tells us nothing about the genetic and environmental origins of obesity as it exists in the world (what is). In the same way, finding that training improves performance (what could be) tells us nothing about the genetic and environmental etiology of existing performance differences in the population (what is). Although there is no necessary relationship between ‘what is’ and ‘what could be’, some of the most far-reaching questions about the acquisition of expertise lie at the interface between ‘what is’ and ‘what could be’, a topic to which we will return in the Discussion.

In relation to the ‘what is’ question, it is a reasonable first step to investigate the extent to which genetic differences contribute to the origins of individual differences in performance because the influence of genetics on individual differences is ubiquitous (Plomin et al., 2013). Genetic research ascribes observed (phenotypic) differences in performance to genetic and environmental components of variance. The proportion of phenotypic variance that can be attributed to genetic differences between individuals is called heritability. Specifically, heritability is a descriptive statistic that describes the average extent to which genetic differences (i.e., differences in DNA sequence) between individuals account for phenotypic differences on a particular measure in a particular sample with its particular mix of genetic and environmental influences at a particular developmental age and secular time (Plomin et al., 2013). In other words, heritability describes ‘what is’ in a particular sample; it does not connote innateness or immutability. Nor does it indicate the mechanisms by which DNA differences affect individual differences in performance. By itself, DNA cannot do anything — it requires an environment inside and outside the body to have its effects. Access to experience and practice is one of the many pathways between genes and behavior. However, the ‘what is’ question is the extent to which differences in such experiences as parenting and practice can account for differences in performance between individuals when controlling for DNA differences between them. Genetically sensitive designs are required to disentangle cause from effect in correlations between experiences and performance.

Even if one believed that expertise is solely due to training and that genetic differences play no role, it would nonetheless be useful to conduct genetic research because it can tell us something important about the source of environmental influence: The extent to which the origins of expertise lie in the family environment. We know that expertise in many domains runs in families but it could do so for reasons of nature or nurture. By controlling for genetic influence, genetically sensitive designs can disentangle nurture from nature. This type of nurture that makes two children growing up in the same family is called shared environmental effects. The surprise from research using genetically sensitive designs in many domains is that shared environmental effects are so small (Plomin, 2011). The environment is important, but the salient environmental effects are not shared by two children growing up in the same family, referred to as nonshared environmental effects. It should be noted that this distinction refers to environmental *effects* on phenotypes, not environmental *events* per se. For example, parental divorce is an environmental event shared by children in the family but divorce could have different effects on the children's adjustment.

In order to provide a concrete example of genetic research on the acquisition of expertise, we investigate reading ‘experts’ — children with exceptional performance on a battery of reading tests such as fluency and comprehension. For the journal *Intelligence*, it might seem odd not to choose as an example exceptional performance on tests of intelligence. We chose reading performance as our example rather than intelligence (or sports, music or games) because reading is a major focus of education in the early school years, which means that all children receive massive amounts of training in reading. In the UK, reading is a key component of the National Curriculum, which dictates specific training in reading that must be accomplished at each stage (Department for Education and Employment, 1999). Another advantage is the evaluation component of the National Curriculum which documents the steep linear increase in reading skills from age 7 to 12 (Department for Education, 2011). In contrast, and unfortunately in our view, schools do not explicitly teach or assess general problem-solving and abstract reasoning, which are the core of intelligence (Jensen, 1998). As discussed later, these differences in the ways in which expertise is acquired for a relatively specific skill like reading as compared to a general ability like intelligence bears importantly on models of the acquisition of expertise.

Much genetic research has examined the normal distribution of individual differences for a wide range of behavioral traits such as cognitive abilities and personality (Plomin et al., 2013). Genetic research has also addressed the low end of performance such as cognitive and learning disabilities, especially reading disability. However, much less is known about the high end of performance, which could have a different etiology from the rest of the distribution. The few genetic studies on high performance, in contrast to normal or low performance, have focused primarily on high intelligence (Haworth et al., 2009). For reading, we are aware of only two twin studies of above-average reading performance. In the first twin study, substantial heritability was reported for 54 MZ and 46 DZ twin pairs for whom at least one co-twin had a reading score more than one standard deviation above the mean from a sample of 350 twin pairs (Boada et al., 2002). The second twin study also reported substantial heritability in an analysis based on a single test of fluency of reading words in the early school years in the same sample as the previous study and in the sample used in the present study; in both studies, children whose reading performance was one standard deviation above the mean were selected (Friend et al., 2009).

The present study extends this previous research in five ways. The previous studies used a selection criterion of reading performance one standard deviation above the mean, which includes the top 15% of the distribution. This modest level of selection was necessitated by the relatively small sample size of the twin sample from which the best readers were selected. The present study began with a sample of more than 10,000 twins (5000 twin pairs), which made it possible to use a cut-off of the top 5% for genetic analyses and to present exploratory results for the top 1%. Second, although investigating the early stages of reading is important, the present study focuses on reading performance at age 12, by which age life-long reading performance is established. Third, the present analyses are based on a composite of four diverse reading measures in order to represent general reading expertise (as in the Boada et al. study) rather than one aspect of reading (as in the Friend et al. study). Fourth, the present study was sufficiently large to explore the relative influence of shared and nonshared environment on reading expertise. Finally, using the same large sample of twins tested at the same age on the same measures, we were able to provide precise comparisons of the etiology of reading expertise and normal reading ability, which will allow us to test an emerging principle from genetic research: The genetic and environmental origins of the high and low extremes of a distribution are the same as the origins of the rest of the distribution (Plomin, Haworth, & Davis, 2009).

## 2 Methods

### 2.1 Participants

The sampling frame for the present analysis was the 12-year assessment wave from the Twins Early Development Study, which recruited families from birth records of twins born in England and Wales in 1994, 1995, and 1996 (Haworth, Davis, & Plomin, 2013). The sample is representative of the UK population ascertained by comparison with census data from the UK Office of National Statistics (Kovas, Haworth, Dale, & Plomin, 2007). Although twins have the option of participating or not during each phase of data collection, the pairs that do participate remain representative of the larger sample. Informed consent was obtained by post or online consent forms, and a test administrator was then assigned who telephoned the family and generally assisted and encouraged. Ethical approval for TEDS was provided by the Institute of Psychiatry ethics committee, reference number 05/Q0706/228.

We excluded from the analyses children with severe current medical problems and children who had suffered severe problems at birth or whose mothers had suffered severe problems during pregnancy. We also excluded twins whose zygosity was unknown or uncertain or whose first language was other than English. Finally, we included only twins whose parents reported their ethnicity as “white”, which is 93% of this UK sample. The present analyses are based on 4955 twin pairs of whom 358 pairs had one twin missing (1788 monozygotic pairs, 1604 same-sex dizygotic, and 1563 opposite-sex dizygotic).

### 2.2 Measures

At age 12, the twins participated in online web-based testing. Widespread access to inexpensive and fast Internet connections in the UK has made online testing an attractive possibility for collecting data on the substantial samples necessary for genetic research, especially for multivariate genetic research. The advantages and potential pitfalls of data collection over the Internet have been reviewed in detail elsewhere (Birnbaum, 2004). For older children, most of whom are competent computer users, it is an interactive and enjoyable medium. Adaptive branching allows the use of hundreds of items to test the full range of ability, while requiring individual children to complete only a relatively small number of items. In tests where it is appropriate, streaming voiceovers can minimize the necessary reading. In addition, the tests can be completed over a period of several weeks, allowing children to pace the activities themselves, although they are not allowed to return to items previously administered. Finally, it is possible to intersperse the activities with games. All of these factors help to maintain children's engagement with the tests. More details about the measures and their psychometric properties are available elsewhere (Haworth et al., 2007).

#### 2.2.1 Reading comprehension

The twins completed an adaptation of the reading comprehension subtest of the Peabody Individual Achievement Test, which assesses literal comprehension of sentences (Markwardt, 1997). The sentences were presented individually on the computer screen. Children were required to read each sentence and were then shown four pictures. They had to select the picture that best matched the sentence they had read, using the mouse. All children started with the same items, but an adaptive algorithm modified item order and test discontinuation depending on the performance of the participant. The Internet adaptation of this test was based on the same instructions, practice items, and test items as the original test.

We also assessed reading comprehension using the GOAL Formative Assessment in Literacy for Key Stage 3 (GOAL plc, 2002). The GOAL is a test of reading achievement that is linked to the literacy goals for children at Key Stage 3 of the National Curriculum. Questions are grouped into three categories: Assessing Knowledge and Understanding (e.g., identifying information, use of punctuation and syntax), Comprehension (e.g., grasping meaning, predicting consequences), and Evaluation and Analysis (e.g., comparing and discriminating between ideas). Within each category, questions about words, sentences, and short paragraphs are asked. Because we were primarily interested in comprehension skills, we used questions from the two relevant categories, Comprehension, and Evaluation and Analysis, with 20 items from each category. Correct answers were summed to give a total comprehension score.

#### 2.2.2 Reading fluency

Reading fluency was assessed using an adaptation of the Woodcock–Johnson III Reading Fluency Test (Woodcock, McGrew, & Mather, 2001) and the Test of Word Reading Efficiency (TOWRE, Form B; Torgesen, Wagner, & Rashotte, 1999). The Woodcock–Johnson test is a measure of reading speed and rate that requires the ability to read and comprehend simple sentences quickly, e.g., “A flower grows in the sky? Yes/No”. The online adaptation consists of 98 yes/no statements; children need to indicate yes or no for each statement as quickly as possible. There is a time limit of 3 min for this test. Correct answers were summed to give a total fluency score.

The TOWRE, a standardized measure of fluency and accuracy in word reading skills, includes two subtests, each printed on a single sheet: a list of 85 words, called Sight-word Efficiency (SWE), which assesses the ability to read aloud real words; and a list of 54 nonwords, called Phonemic Decoding Efficiency (PDE), which assesses the ability to read aloud pronounceable printed nonwords. The child is given 45 s to read as many words as possible. Twins were individually assessed by telephone using test stimuli that had been posted to families in a sealed package with separate instructions that the package should not be opened until the time of testing. The same tester, who was blind to zygosity, assessed both twins in a pair within the same test session.

Although separate analyses of each of the four reading tests, and multivariate analyses among them, could be interesting, we will present results for a general composite of the four reading tests in order to simplify the presentation and for two psychometric reasons: The four tests load highly (0.56–0.77) on a first unrotated principal component, and the composite score is more reliable than the individual test scores. The composite was constructed by standardizing each of the four scores and summing them so that the four measures were equally weighted. At least three of the four measures were required to be non-missing. The composite score was also standardized.

### 2.3 Analyses

Three types of twin analyses were used in this study: the standard univariate twin model-fitting analysis of individual differences in reading performance for the total sample, liability-threshold model-fitting using dichotomous data (expert versus not expert), and DeFries–Fulker (DF) extremes analysis which selects an extreme group (experts) and analyzes quantitative trait variation in co-twins of expert readers.

#### 2.3.1 Twin model-fitting analysis

According to the quantitative genetic model (Plomin et al., 2013), twins reared together resemble each other due to the additive effects of shared genes (A) or shared (common) environmental factors (C). For identical or monozygotic (MZ) twins, the correlation between their genes is 1.00, whereas for nonidentical or dizygotic (DZ) twins, the correlation is .50 because DZ twins on average share half of their segregating alleles. The correlation between twins for shared environment is, by definition, 1.00 for both MZ and DZ twins growing up in the same family, whereas nonshared environmental influences (E) are uncorrelated and contribute to differences between twins.

OpenMx software for structural equation modeling was used to perform standard model-fitting analyses using raw data (Boker et al., 2011). Two fit indices were considered: Chi-square and Akaike's information criterion, AIC (Akaike, 1987). For the twin analyses, standardized residuals correcting for age and sex were used because the age of twins is perfectly correlated across pairs, which means that, unless corrected, variation within each age group at the time of testing would contribute to the correlation between twins and be misrepresented as shared environmental influence. The same applies to the sex of the twins, since MZ twins are always of the same sex but only half of DZ twin pairs are of the same sex. The assumptions of the classical twin model, and their validity, have been discussed in detail elsewhere (Boomsma, Busjahn, & Peltonen, 2002; Visscher, Hill, & Wray, 2008).

#### 2.3.2 Liability-threshold model-fitting using dichotomous data

The dichotomous data for experts versus non-experts can be examined by comparing twin concordance for MZ and DZ twins. In the Results section, two types of twin concordance are calculated. The most intuitive concordance is pairwise concordance, which is the number of concordant pairs divided by the number of total concordant plus discordant pairs. However, a less obvious index of twin concordance is preferred because it indicates morbidity risk, the chance that a cotwin of an expert will also be an expert. This index, called probandwise concordance, is the number of individual twins in concordant pairs divided by the total number of individual twins.

Dichotomous data such as diagnoses of disorders are often analyzed using a liability-threshold model that assumes that liability is distributed normally but the disorder occurs only when a certain threshold of liability is exceeded. We apply this model and analysis to expert readers. The dichotomous data (expert vs non-expert) were used to calculate tetrachoric twin correlations and thresholds (Falconer, 1965; Smith, 1974) and perform standard liability-threshold modeling using openMx (Boker et al., 2011).

Model-fitting analyses estimate analogous ACE parameters as in twin model-fitting analyses of continuous data. However, the heritability estimate derived from liability-threshold model-fitting is not the heritability of expertise as assessed quantitatively; it is the heritability of a hypothetical construct of continuous liability derived from dichotomous data.

#### 2.3.3 DeFries–Fulker (DF) extremes analysis

If the only available data were a ‘diagnosis’ of expertise, the liability-threshold modeling is a useful way of assuming an underlying continuous liability despite having assessed a dichotomy. However, analyzing expertise as a dichotomy loses much information, especially in light of the fact that we have assessed expertise as a continuum. Capitalizing on our design that begins with a representative sample and uses a quantitative measure that assesses continuous variation in reading performance, an analysis called DF extremes analysis (DeFries & Fulker, 1985, 1988; DeFries, Fulker, & LaBuda, 1987), makes use of the quantitative trait data to estimate the genetic and environmental origins of the difference in mean performance between the ‘experts’ and the rest of the population. DF extremes analysis assesses the extent to which the quantitative trait scores of co-twins of the extreme group (experts in this case) regress back to the population mean on the quantitative trait. If the mean of the co-twins is the same as the population mean, there is no familial resemblance. Comparing the regression to the mean for MZ and DZ co-twins of the expert group indicates genetic influence on the mean difference between the experts and the population on the quantitative trait score.

In DF extremes analysis, quantitative trait scores are standardized and transformed to adjust for proband mean differences between MZ and DZ groups so that genetic and environmental parameters can be estimated from structural equation model fitting based on linear regression:

$$C\left(X\right)=B1P\left(X\right)+B2R+A\text{,}$$

where the co-twin's reading score, *C*(*X*), is predicted from the proband's reading score, *P*(*X*), and the coefficient of relatedness (*R*), which is 1.0 for MZ twins (who are genetically identical) and 0.5 for DZ twins (who are on average 50% similar genetically for additive genetic effects). The regression weight *B*2 estimates group heritability because it tests whether the proband and co-twin mean differ for MZ and DZ twins (genetic relatedness, *R*). This heritability is called *group* heritability as it refers to genetic influence on the mean quantitative trait score difference between the proband group and the population, in contrast to the usual estimate of heritability, which could be called *individual differences* heritability because it refers to genetic influence on individual differences throughout the distribution.

## 3 Results

The goal of our study is to test the reasonable hypothesis that expert reading performance is due solely to environmental factors, or whether genetic factors also contribute to reading expertise. The twin method also makes it possible to test the hypothesis that the salient environmental influences are factors such as parents and schools that are shared by children growing up in the same family and attending the same schools. This section reports the results of twin analyses of the reading performance of experts and non-experts, including analyses of dichotomous data (expert versus not expert) using liability threshold model-fitting and analyses that incorporate quantitative trait data on reading performance using DF extremes analysis. We also apply the classical twin model-fitting analysis to individual differences within the expert group and within the total sample. We begin with a description of the distribution and our rationale for selecting expert readers.

### 3.1 Descriptive results

**Fig. 1:**
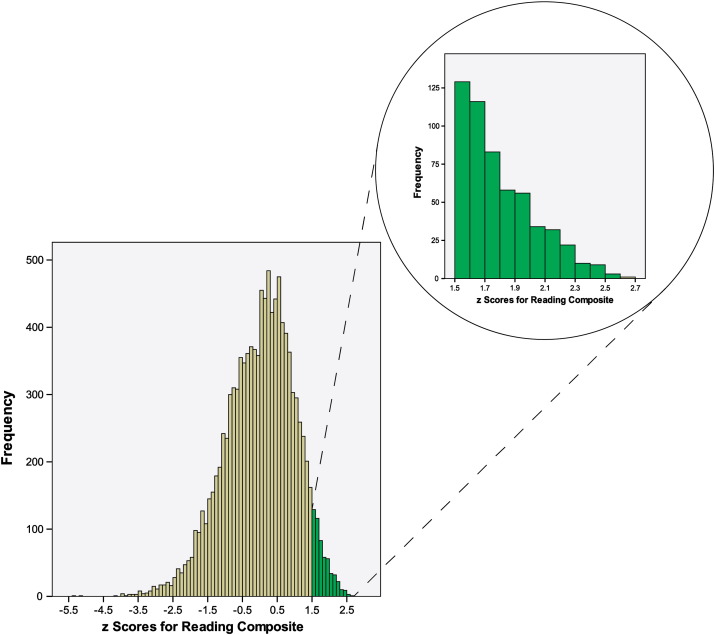
Distribution of standardized composite reading scores at age 12. N = 10,698. We operationally define as ‘expert readers’ the 506 children who scored 1.5 standard deviations above the mean.

Basic descriptive statistics for the four reading measures are available elsewhere (Davis, Haworth, & Plomin, 2009). An analysis of variance testing the effects of zygosity and sex found that these accounted for less than 1% of the variance for each of the four measures. Fig. 1 shows the distribution for 10,698 individuals' standard (z) scores on the composite reading measure at age 12. Although the scores are reasonably normally distributed, there are fewer very high scores than very low scores. This negative skewness was caused by ceiling effects and a large low-performance tail for the reading tests, especially the two reading comprehension tests, which reflects the goal of the reading battery, which was to differentiate low performance rather than high performance. Nonetheless, when we operationally defined as a cut-off for reading expertise the top 5% of the distribution (N = 544 with standard scores greater than 1.5 standard deviations above the population mean), the average score of this group is 1.8 standard deviations above the mean. This cut-off represents a trade-off between level of expertise and power of the genetic analysis. Although higher levels of performance could yield different results, the group of 107 individuals in the top 1% of the distribution (2.0 standard deviations above the population mean) is not sufficiently large to provide adequately powered genetic analyses, although we will mention results for this more exceptional group. Even using the top 5% of the distribution as a cut-off for expertise, we had insufficient power in our genetic analyses to detect sex differences in expertise, and as a result we conducted analyses for the entire sample rather than subdividing the sample by sex.

The primary aim of our study is to estimate the genetic and environmental origins of the 1.8 standard deviation difference between the ‘experts’ and the rest of the population, which we refer to as the ‘between-group’ difference. The enlarged portion of Fig. 1 focuses on the distribution of the 506 expert readers. Although these individual differences within the expert group are small compared to the average difference between the expert group and the rest of the distribution, we can also investigate the genetic and environmental origins of individual differences within the expert group.

### 3.2 To what extent is the average difference between expert readers and the rest of the sample due to genetic and environmental influences?

#### 3.2.1 Twin concordances using dichotomous data

The twin method can address several questions about the genetic and environmental etiology of expertise. Most of these questions rely on the dichotomy of expert versus not expert. Fig. 1 shows the distribution of all individuals in the sample, including both members of twin pairs. In order to avoid biased sampling for analyses of twin concordances, we randomly selected one member of each twin pair and re-selected the top 5% from this group, which resulted in 321 expert readers and 167 non-expert readers (488 individuals) in 244 twin pairs (66 MZ pairs and 178 DZ pairs) with complete data. (Note: The full sample of 4955 twin pairs was used for all twin analyses other than concordances including tetrachoric correlations, liability-threshold and DF extremes). As indicated in Methods, the simplest method to analyze these dichotomous data (i.e., expert versus not expert) is to calculate twin concordance, the presence of expertise in both members of a twin pair. The 132 MZ twins included 70 twins in 35 concordant pairs plus 62 twins in 31 discordant pairs; the 356 DZ twins included 84 twins in 42 concordant pairs plus 272 twins in 136 discordant pairs. The simplest index of twin concordance, called pairwise concordance, is the number of concordant pairs divided by the number of total concordant plus discordant pairs, which is 53% for MZ twins (35/66) and 24% for DZ twins (42/178). Because the MZ twin concordance is more than twice as great as the DZ twin concordance, this result suggests genetic influence.

As indicated in the Methods section, probandwise concordance is preferable. The probandwise twin concordances for reading expertise are also 69% for MZ twins (70/101) and 38% for DZ twins (84/220). In other words, if a child were an expert reader, the chance that the child's twin would also be an expert reader is about 70% for MZ twins and about 40% for DZ twins. Because the concordance for MZ twins is twice the concordance for DZ twins, this result suggests genetic influence on reading expertise. Doubling the difference in MZ and DZ twin concordances suggests a heritability of about 60%. The use of concordances for this purpose is not completely appropriate statistically because, unlike tetrachoric correlations and intraclass correlations (discussed below), concordances do not incorporate population base rates, although this problem primarily affects estimates of shared environment rather than estimates of heritability. Because MZ twin concordance is 70% and heritability is only 60%, the excess MZ resemblance not accounted for by heritability (10%) could be ascribed to shared environment, minus the 5% base rate used to select experts in this study.

What about more exceptional performance than the top 5%? As mentioned earlier, the current sample size of more than 10,000 twin children is not large enough to produce adequate power for genetic analyses for the top 1%, which includes only 112 individuals. Nonetheless, the results for this top 1% group are similar to the results found using a 5% cut-off: Probandwise concordance was 50% for MZ twins and 18% for DZ twins, suggesting about 50% heritability (which cannot exceed MZ similarity) and no shared environmental influence.

#### 3.2.2 Liability-threshold model-fitting using dichotomous data

As indicated in Methods, dichotomous data such as diagnoses of disorders are often analyzed using a liability-threshold model that assumes that liability is distributed normally but expertise occurs only when a certain threshold of liability is exceeded. The liability-threshold model is based on twin tetrachoric correlations derived from dichotomous data. Using our dichotomous data on reading expertise, the twin tetrachoric correlations are 0.87 (.03 SE) for MZ twins and 0.48 (.05 SE) for DZ twins. These twin tetrachoric correlations can then be analyzed in the same way as twin correlations based on continuous data — for example, doubling the difference between MZ and DZ correlations to estimate heritability. This rough estimate of heritability is 76%, which is similar to the liability-threshold model-fitting estimate of 75%, with a 95% confidence interval (CI) from 0.53 to 0.92. As noted in Methods, however, this heritability estimate based on dichotomous data is not the heritability of expertise as assessed quantitatively; it is the heritability of a hypothetical construct of continuous liability derived from dichotomous data. Similar to the analyses described above which were based on concordances, shared environmental influence is negligible based on these twin tetrachoric correlations (.10), the same as the liability-threshold model-fitting estimate, which is not significant, as indicated by its 95% confidence interval which included zero (.00–.30).

#### 3.2.3 DeFries–Fulker (DF) extremes analysis

As described in Methods, DF extremes analysis uses quantitative trait data to estimate the genetic and environmental origins of the 1.8 standard deviation difference in mean performance between the ‘experts’ and the rest of the population. As indicated in Methods, DF extremes analysis assesses the extent to which the quantitative trait scores of co-twins of the extreme group (experts in this case) regress back to the population mean on the quantitative trait. In order to illustrate the DF extremes analysis, we randomly selected only one member of twin pairs in the top 5% as experts, which reduced the sample of experts to 244.

**Fig. 2:**
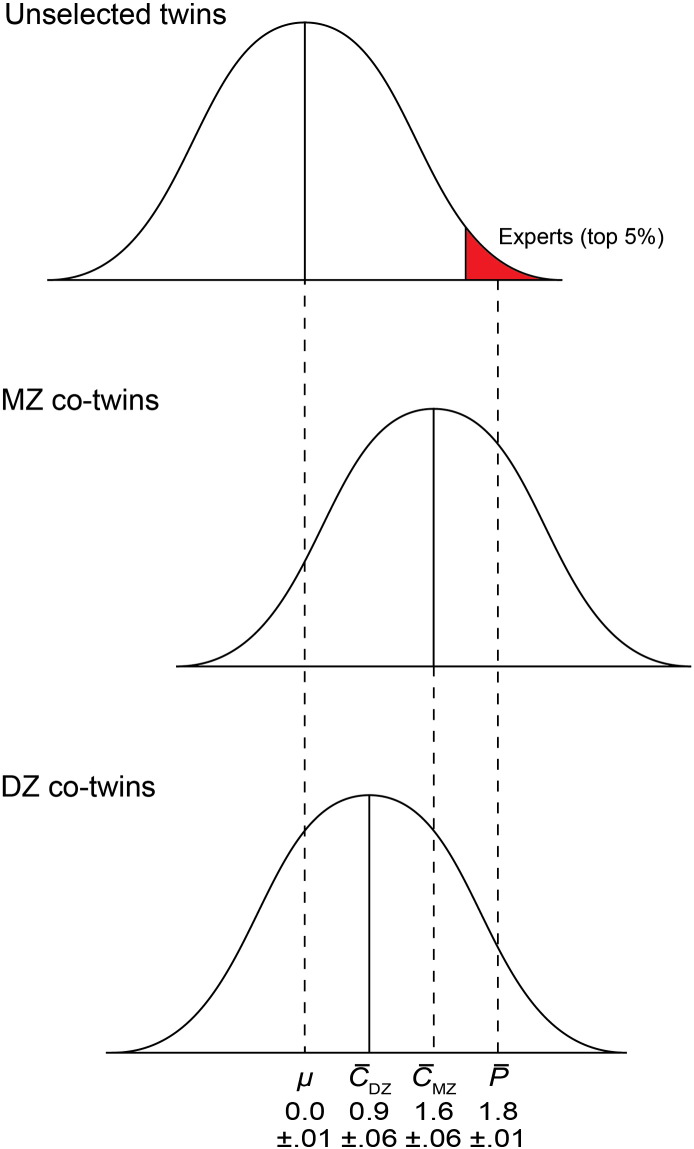
DF extremes analysis: Investigating the etiology of expertise by comparing the regression to the population mean for MZ and DZ co-twins of experts in the top 5% of reading performance. The MZ co-twins resemble the experts in that their mean reading score does not regress very far back to the population mean. In contrast, DZ co-twins regress halfway back to the population mean. See text for explanation and interpretation.

Fig. 2 shows that after selecting the top 5% of the total unselected sample of twins as experts, the MZ co-twins of experts regress less far back to the population mean as compared to the DZ co-twins of experts on our quantitative trait measure of reading. This result suggests genetic influence because the co-twins' scores are more similar to the probands for MZ as compared to DZ co-twins. The mean standard score of the experts is 1.8, with a standard error of ± .02 (the standard deviation divided by the square root of N, which is 244). The population mean is 0.0 (± .01, N = 10,698). The mean of all the co-twins of the experts is 1.1 (± .05, N = 244), suggesting familial resemblance in that the co-twins regress less than halfway back to the population mean of 0.0 from the expert mean of 1.8. The mean of MZ co-twins is 1.6 (± .07, N = 66), close to the expert mean of 1.8. The mean of DZ co-twins is 0.9 (± .06, N = 178), regressing halfway back to the population mean of 0.0.

This differential regression to the mean for MZ and DZ co-twins estimates genetic influence and can be quantified by calculating a twin ‘group’ correlation, which can be interpreted like a typical twin correlation except that the twin group correlation refers to the average difference between the extreme group and the rest of the population rather than to individual differences in the population (Plomin, 1991). The design shown in Fig. 2 is like a selection design in animal studies in the sense that studies of artificial selection compare ‘response to selection’ (the mean difference between the cotwins and the population) to the ‘selection differential’ (the mean difference between the probands and the population). The ratio between response to selection and selection differential is used in selection studies to estimate ‘realized heritability’. The same ratio can be used in twin studies to estimate group correlations. The numerator of the ratio (response to selection) is 1.6 for MZ co-twins (i.e., 1.6–0.0) and 0.9 for DZ co-twins (i.e., 0.9–0.0); the denominator (selection differential) is 1.8 for both MZ and DZ twins (1.8–0.0). Thus, the group correlation is 0.89 for MZ twins (i.e., 1.6/1.8) and 0.50 for DZ twins (0.9/1.8). Doubling the difference in these MZ and DZ group correlations estimates a ‘group’ heritability of 78%, indicating that genetics accounts for about three-quarters of the mean difference between the experts and the population. This group heritability estimate is similar to the estimate of 66% (.49–.83 CI) from a DF extremes model-fitting analysis, even though the DF extremes analysis takes into account cases in which both members of a twin pair are in the expert group rather than randomly selecting one member of such pairs as we have done in the illustrative analyses above. DF extremes analysis also takes into account mean differences between the MZ and DZ probands, but these means are the same (1.8) in our study. Similar to the previous analyses of dichotomous data, MZ and DZ group correlations suggest little role for shared environmental influence in the sense that group heritability of 0.78 is nearly as high as the MZ group correlation of 0.83. This residual MZ twin resemblance of 0.05 is ascribed to shared environmental influence; the estimate of shared environmental influence from DF extremes model-fitting analysis is somewhat higher, 0.17 (.06–.29 CI).

The group heritability estimate of 66% from DF extremes analysis is similar to the analyses of dichotomous data described above for liability-threshold model-fitting (75%). If the assumptions of the liability-threshold model-fitting are correct, its results should approximate those from DF extremes analysis (Plomin & Kovas, 2005). Also noteworthy is the finding that, despite the very small sample sizes for the 1% cut-off (N = 18 MZ and 38 DZ experts), the results are quite similar for this considerably more exceptional group, with MZ and DZ group correlations of .89 and .53, suggesting a DF extremes group heritability of 72%.

In summary, all of the genetic analyses suggest that genetics accounts for more than half of the mean difference in reading performance between the experts and the rest of the distribution. Shared environmental factors are similarly modest (less than 20%) for both DF extremes analysis of quantitative data and liability-threshold model-fitting.

### 3.3 To what extent are individual differences within the group of expert readers due to genetic and environmental influences?

Another conceptually very different analysis of expertise focuses on individual differences within the group of experts. Confronted with quantitative trait data from twins in which at least one co-twin is an expert, the most obvious thing to do is to calculate MZ and DZ twin correlations. However, this is a very different question because it ignores the large performance difference between experts and the population and focuses on the relatively small individual differences within the expert group. Moreover, conducting individual differences analyses within a highly selected group introduces the problems of restriction of range and a non-normal distribution (see the expanded panel of Fig. 1). Because scores were standardized, the standard deviation for the total sample was 1.0 (Fig. 1). In contrast, using a 5% cut-off for expertise, the standard deviation for this expert group is reduced to 0.25. Nonetheless, ignoring this restriction of range and non-normal distribution, the twin correlations for the 244 twin pairs in which at least one co-twin was an expert were 0.44 for MZ twins and 0.22 for DZ twins. Doubling this difference between MZ and DZ twin correlations estimates heritability as 44%. Shared environmental influence is negligible (0%). Model-fitting analyses yield similar estimates of heritability (.41; .10–.61 CI) and shared environment (.00; .00–.14 CI).

It should be emphasized that these estimates of genetic and shared environmental influence refer to the relatively small individual differences in reading performance within the expert group. Nonetheless, it is interesting to note that genetics accounts for about half the individual differences within the expert group, as well as about half the average difference between the expert group and the rest of the population. What about individual differences across the entire distribution? This is no longer an analysis of reading expertise per se but rather it is about individual differences in reading performance throughout the distribution, from poor readers to expert readers. Twin correlations for the entire sample are 0.76 for MZ twins and 0.45 for DZ twins. Doubling this difference in twin correlations estimates heritability as 62%. Shared environmental influence is estimated as 14% (i.e., 0.76–0.62). ACE model-fitting analyses yielded similar estimates of heritability (.62; .56–.69 CI) and shared environment (.14; .08–.19 CI).

## 4 Discussion

It is not unreasonable to hypothesize that expertise is due solely to environmental factors, not genetics:

“Many characteristics once believed to reflect innate talent are actually the result of intense practice extended for a minimum of 10 years” (Ericsson, Krampe, & Tesch-Romer, 1993).

“Differences in early experiences, preferences, opportunities, habits, training, and practice are the real determinants of excellence” (Howe et al., 1998).

“It is possible to account for the development of elite performance among healthy children without recourse to unique talent (genetic endowment) — excepting the innate determinants of body size” (Ericsson, 2007).

However, when we put this environmental hypothesis to the test, it fails badly: No matter how we analyzed the data, the results indicated that more than half of the difference in performance between expert readers and normal readers is due to genetic factors. A recent meta-analysis of research on the correlation between deliberate practice and expertise in chess and music, found that practice accounted for only about a third of the variance after accounting for unreliability of measurement (Hambrick et al., 2013).

It is also not unreasonable to hypothesize that the salient environmental influences are factors such as parents and schools that are shared by children growing up in the same family and attending the same schools: “The theoretical framework of expert performance explains individual differences in attained performance by the factors that influence the engagement in sustained extended deliberate practice, such as motivation, parental support, and access to the best training environments and teachers” (Ericsson, 2007). However, when we test this hypothesis of shared environmental influence, it also fails badly: Less than a fifth of the difference in performance between experts and the rest of the population can be explained by shared environmental factors such as growing up in the same family or attending the same schools. Moreover, it is likely that our twin study estimates of shared environment are inflated in the sense that they include experiences shared by twins who are exactly the same age and who grow up simultaneously in the same womb, same home, and same school (Koeppen-Schomerus, Spinath, & Plomin, 2003).

As indicated in the Introduction, it is important to emphasize that genetic research addresses the ‘what is’ question — the effect of DNA sequence variation on performance differences as they are assessed in a particular population at a particular time with that population's particular mix of genes and environments (including training). Genetic research does not address the ‘what could be’ question — if you change the environment by providing a new training program you could change reading performance. We hope that this example of the acquisition of reading expertise shows why it is wrong to talk about genetic influence in terms of “genetic constraints” and “heritable limits” (Ericsson, 2007) — this confuses ‘what is’ and ‘what could be’.

It should be noted that our study has several limitations. First, there are possible limitations of the twin method such as the equal environment assumption (Plomin et al., 2013), although it is noteworthy that similar results suggesting substantial heritability for individual differences in reading performance have been found for a completely different design, a parent–offspring adoption study (Wadsworth, Corley, Hewitt, Plomin, & DeFries, 2002). An obvious limitation specific to our study is the use of a 5% cut-off rather than a more exceptional level of performance. As noted earlier, our cut-off of 5% represented a balance between extreme performance and sample sizes needed for adequately powered genetic analyses. We acknowledge that results might well differ for more extreme cut-offs, although our exploratory analyses using a 1% cut-off yielded similar results. A second limitation is that we focused on a single measure (a composite measure of reading) at a single age (12 years). Many interesting questions lie in multivariate issues (e.g., the etiological links between reading expertise and other cognitive abilities) and developmental issues (e.g., the etiological links between reading expertise at age 12 and reading performance at earlier ages). Our decision to focus on a single measure at a single age followed from the goal of this paper which was to use reading performance at age 12 merely as an example of issues of nature and nurture in the acquisition of expertise.

### 4.1 What is inherited is DNA sequence variation

There is confusion about how inherited DNA sequence variation relates to gene expression, which involves the transcription of DNA to RNA. Although there are examples of this confusion in many domains, an example from the domain of expertise is the argument that genetic research does “not offer complete genetic accounts that specify the causal processes involved in the activation and expression of the dormant genes in DNA during practice” and that “all healthy individuals seem to have the critical genes required for the desired changes as part of their cells' dormant DNA” (Ericsson, 2007, pages 4 and 27). This suggestion that practice drives genetics by activating and expressing ‘dormant’ DNA misses the point that all that is inherited is DNA sequence variation. The DNA sequence in the single cell with which your life began is the same DNA sequence in all of the trillions of cells in your body for the rest of your life. Nothing changes your DNA sequence variation — not environment, biology or behavior. What changes is the rate of transcription of your DNA sequence into RNA. For example, you are changing the transcription of your DNA that codes for neurotransmitters as you read this sentence. If your inherited DNA sequence coding for one of these neurotransmitters differs functionally from other individuals, this coding difference will appear every time that your DNA is transcribed into RNA — as you read, think and practice. Transcription of DNA into RNA is a response to the environment; what is inherited is DNA sequence variation.

All of the other -omics in between genomics and behavior – epigenomics, transcriptomics, proteomics – are important for understanding pathways between genes and individual differences in outcomes, but they are not inherited from parent to offspring (Plomin, 2013). That is, all that is inherited is DNA sequence variation — everything else is a phenotype. For this reason, DNA sequence variation is in a causal class of its own in the sense that there is no direction of effects issue when it comes to correlations between genes and behavior. In other words, correlations between DNA sequence variation and behavior are ultimately causal from genes to behavior because our behavior and experiences do not change DNA sequence variation. Other correlations between behavior and biology, including all the -omics and the brain, raise questions about the direction of effects, that is, whether the correlation is caused by the effects of behavior on biology or vice versa.

Although we have focused on quantitative genetic research using the twin design, the future of behavioral genetics lies in molecular genetic research using DNA (Plomin, 2013). Although current attempts to identify specific genes have had only limited success, known as the missing heritability problem, whole-genome sequencing will improve this situation by identifying all DNA sequence variation, including rare variants. Because the heritability of complex traits is caused by many DNA variants of small effect in the population, polygenic scores that are composites of hundreds or thousands of DNA variants will be used to predict genetic propensities for expert performance. The most far-reaching advance will be the widespread availability of whole-genome sequence for children, which means that researchers would no longer need to obtain DNA or to genotype children in order to use genomic information in research (Plomin & Simpson, in press).

### 4.2 The abnormal is normal

In addition to documenting the important role of genetics in expert reading, the results broach the important issue of the etiological links between the abnormal (expert extremes of performance) and the normal distribution of individual differences. Is expertise special or even unique etiologically? The acquisition of expertise could be due to special environmental and genetic factors that do not affect performance in the normal range. For example, special training opportunities might be a source of environmental influence specific to expertise; it would be reasonable to expect that children who become expert readers might have had special training from parents and teachers. Less obvious is a genetic hypothesis called *emergenesis* that postulates genetic factors unique to expertise; it is an extreme form of nonadditive (epistatic) genetic influence in which rare combinations of many genes are responsible for exceptional performance (Lykken, 2006).

In principle, we could have found that the genetic and environmental etiology of expertise was different from the rest of the distribution, but instead we found that their etiologies are highly similar in the case of reading. DF extremes analysis of the expert group yielded 66% group heritability and 17% group shared environment, and individual differences analysis for the entire sample yielded 62% individual differences heritability and 14% individual differences shared environment. The similarity of these results is likely to be more than coincidental: The results are consistent with the hypothesis that the same substantial genetic factors and modest shared environmental factors are responsible for expertise in reading and for individual differences in reading performance throughout the distribution. Stated more provocatively, reading expertise might be nothing more than the quantitative extreme of the same genetic and environmental factors responsible for normal variation in reading.

Support for this abnormal-is-normal hypothesis comes from a more subtle interpretation of DF extremes analysis. Because DF extremes analysis is based on the link between an extreme group (e.g., reading experts) and a quantitative trait (e.g., reading performance in the total sample), finding substantial group heritability not only implies that both the extreme group and the quantitative trait are heritable, but also that there are strong genetic links between them. In other words, the genetic correlation between them must be high, suggesting that the same genes affect the average difference between the expert group and the population on the one hand and individual differences within the population on the other (Plomin & Kovas, 2005). The strongest test of this abnormal-is-normal hypothesis will come when specific genes are identified that account for some of the heritability of individual differences in reading performance (Plomin & Simpson, in press): We predict that the same genes will be associated with the difference between expert and normal readers.

The abnormal-is-normal hypothesis appears to hold generally — not just for behavioral traits such as learning abilities and disabilities (Plomin & Kovas, 2005) but also for medical disorders (Plomin et al., 2009). For this reason, this hypothesis could be viewed as the default prediction for the acquisition of expertise in other domains that have not been as well studied genetically, such as music, sports and games.

### 4.3 The nature of nurture: from a passive model of imposed environments to an active model of shaped experience

Although there is no necessary relationship between ‘what is’ and ‘what could be’, some of the most far-reaching questions about the acquisition of expertise lie at the developmental interface between them. We focus on childhood because reading expertise has its roots in childhood, as does exceptional performance in most domains. Experiments on ‘what could be’ are embedded in a passive model of the environment in which a training regime is imposed on children. A passive model of training assumes a one-size-fits-all approach to the acquisition of experience.

#### 4.3.1 Genotype–environment interaction

One way to incorporate genetics into this passive model is to consider genotype–environment interaction, which denotes genetically driven sensitivity to an imposed environment (Kendler & Eaves, 1986; Plomin, DeFries, & Loehlin, 1977). In other words, the effect of the environment on a phenotype depends on genotype. In relation to the acquisition of expertise, genotype–environment interaction refers to the possibility that children respond differently to a training regime on the basis of genetic differences between them. Research on gene-by-environment interaction is growing rapidly as researchers increasingly incorporate DNA in their research (Karg, Burmeister, Shedden, & Sen, 2011). For example, DNA research on sports performance is burgeoning (Sawczuk, Maciejewska, Cieszczyk, & Eider, 2011). For exceptional athletic performance, a meta-analysis of 366 studies found that a polymorphism in the angiotensin I-converting enzyme gene (*ACE*) is significantly associated with performance in endurance athletes, and a meta-analysis of 88 studies found that a polymorphism in the alpha-actinin-3 gene (*ACTN3*) is associated with power events (Ma et al., 2013). The greatest growth area is in pharmacogenetics, the genetics of individual differences in response to drugs (Ma & Lu, 2011).

We predict that candidate gene research on interactions with expert training will blossom. However, caution is warranted because a meta-analysis of more than 100 gene-by-environment interactions shows that most such reports do not replicate (Duncan & Keller, 2011), and some journals are now requiring independent replication for publication of gene-by-environment interactions findings (Hewitt, 2012). On the other hand, we note that these published gene-by-environment interaction studies assessed naturally occurring environments such as parenting. A possible advantage of expert training studies of gene-by-environment interaction is that a more specific, uniform, and powerful training regime can be imposed experimentally to assess the extent to which responses to training are moderated genetically (van Ijzendoorn et al., 2011).

#### 4.3.2 Genetic research on training

**Fig. 3:**
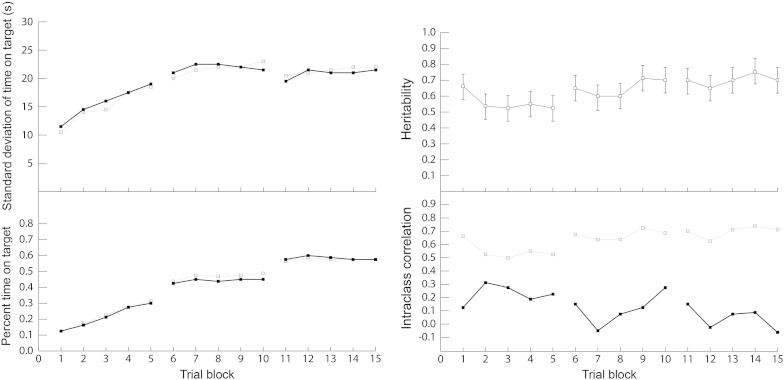
A twin study of initial proficiency and acquisition of expertise on a motor-skill task with feedback given over 5 blocks of trials on each of 3 days. Open squares indicate monozygotic twins, closed squares indicate dizygotic twins.

Adapted from Fox et al. (1996).

Another way in which genetics can be incorporated in training research is to use genetic methods to investigate the genetic and environmental origins of individual differences in the process of training rather than the outcomes of training. There are very few quantitative genetic studies that have incorporated behavioral interventions (Plomin & Haworth, 2010). Even in the well studied domain of cognitive abilities, there are few human studies of the learning process. One instructive example is a study of motor learning given over five blocks of trials on each of three days (Fox, Hershberger, & Bouchard, 1996). Although the sample size was small, the design was the powerful twins reared-apart design, with 64 pairs of identical twins reared apart (MZA) and 32 pairs of fraternal twins reared apart (DZA). As shown in Fig. 3 (lower left), both MZA and DZA twins acquired skill during 15 blocks of trials administered over three days, from about 15% time on target in initial trials to about 60% time on target in later trials. From an individual differences perspective, it is interesting that variance also increased during training, indicating that some twins improved more than others (upper left of Fig. 3). The MZ and DZ correlations (lower right) and heritability estimates (upper right) indicate that genetic influence on individual differences in acquisition of this motor skill is substantial not only at the beginning of training, but also during training and at the end of training. There is even a suggestion that heritability increases as a result of training, from about 55% to about 70%. Another interesting result of this study is that individual differences in change in performance as assessed by each individual's slope within each block of five trials also showed high heritability, suggesting genetic influence on individual differences in acquisition.

The authors end their article with an important distinction between mean performance and individual differences in performance:

“This conclusion [about the importance of genetics] does not diminish the importance of practice with feedback for the acquisition of skill. Even the least gifted of our twins attained levels of skill after practice that were superior to those achieved in initial trials by the most gifted.” (Fox et al., 1996, p. 357.)

Much more information can be mined from training studies embedded in genetically sensitive designs by using longitudinal genetic analysis techniques that can assess acquisition and post-training performance independent of pre-training performance (Plomin & Haworth, 2010).

#### 4.3.3 Genotype–environment correlation

Although gene-by-environment interaction research incorporates genetics in studies of expertise training, it accepts the passive environmental model in which a training regime is imposed from the outside on children. That is, interaction studies investigate the extent to which the effect of an imposed environment on children depends on genotype. In contrast, a very different and more fundamental way of thinking about the interplay between genes and environment in relation to the acquisition of expertise is genotype–environment correlation rather than interaction — mediation rather than moderation. Genotype–environment correlation has been described as genetic control of exposure to the environment, in contrast to genotype–environment interaction, which involves genetic sensitivity to imposed environments (Kendler & Eaves, 1986).

The importance of genotype–environment correlation became clear in the 1980s when it was found that most measures ostensibly assessing psychologically relevant aspects of the environment such as parenting and life events in fact show substantial genetic influence (Knafo & Jaffee, 2013; Plomin & Bergeman, 1991). Of course, environments per se cannot show genetic influence. However, measures of the environment can show genetic influence to the extent that they are correlated genetically with behavioral traits. For example, life events have been used as environmental measures in thousands of studies, but life events are not measures of an objective environment ‘out there’ that happens passively to people. The likelihood that we will experience problems with relationships, financial disruption, and other life events – and nearly all other environmental measures used in psychological research – depends in part on genetically influenced behavioral traits (McAdams, Gregory, & Eley, 2013). A review of 55 independent genetic studies using environmental measures found an average heritability of 27% across 35 different environmental measures (Kendler & Baker, 2007). There are few measures of psychologically relevant environments that do not show genetic influence when investigated in adequately powered genetically sensitive studies; significant genetic influence has been reported for some unlikely experiences such as childhood accidents (Phillips & Matheny, 1995), bullying victimization (Bowes et al., 2013), and children's television viewing (Plomin, Corley, DeFries, & Fulker, 1990). Although genotype–environment correlation has not been studied explicitly in relation to the acquisition of expertise, it is safe to assume that putative environmental factors associated with the development of expertise will also show genetic influence. For example, parenting is often mentioned as an environmental factor in the origins of expertise, but nearly all aspects of parenting consistently show genetic influence (Plomin et al., 2013).

How do genetic factors contribute to variations in environments that we experience? There are three types of genotype–environment correlation: passive, evocative, and active (Plomin et al., 1977). The passive type occurs when children passively inherit from their parents' family environments that are correlated with their genetic propensities. The evocative type happens when individuals, on the basis of their genetic propensities, evoke reactions from other people on the basis of their genetic propensities. The active type emerges when individuals shape their own experiences in ways correlated with their genetic propensities, which include appetites (motivation) as well as aptitudes.

For example, consider the development of expertise in reading. Children who are expert readers are likely to have parents who read well and provide their children with both genes and an environment conducive to the development of reading (passive genotype–environment correlation). Children with a genetic propensity towards reading might also be picked out at school and given special opportunities (evocative type). Even if no one does anything about their reading, children with genetic proclivities towards reading can seek out their own enriched reading environments, for example, by selecting friends who like to read, or simply by reading more books (active type). We suggest that such genotype–environment correlational processes are important mechanisms by which children develop expertise in other domains as well, such as sports and music.

The ramifications of genotype–environment correlation go far beyond demonstrating that so-called environmental measures include substantial genetic influence. It represents a general view of the way genotypes become phenotypes (Plomin, 1994). Specifically in relation to the development of expertise, genotype–environment correlation research leads to an active model of experience in which children select, modify, and create their own environments in part on the basis of their genetic propensities. Rather than thinking about the development of expertise as the passive acquisition of an imposed one-size-fits-all training regime, this active model of genetically guided experience leads to a more individualized approach. The essence of the active model of experience is choice — allowing children to sample an extensive menu of experiences so that they can discover their appetites as well as aptitudes. This active model of genotype–environment correlation might be more cost-effective in fostering expertise than the passive training model – and it will certainly be more fun for parents as well as children – because if all goes well, children will try to become the best they can be because they want to, not because they are made to do it.
